# Spatiotemporal Distribution of Agrin after Intrathecal Injection and Its Protective Role in Cerebral Ischemia/Reperfusion Injury

**DOI:** 10.1002/advs.201902600

**Published:** 2019-12-19

**Authors:** Shiyong Li, Ye Wang, Dawei Jiang, Dalong Ni, Christopher J. Kutyreff, Todd E. Barnhart, Jonathan W. Engle, Weibo Cai

**Affiliations:** ^1^ Department of Rehabilitation Second Affiliated Hospital of Nanchang University Nanchang Jiangxi 330006 China; ^2^ Department of Neurology Second Affiliated Hospital of Nanchang University Nanchang Jiangxi 330006 China; ^3^ Departments of Radiology and Medical Physics University of Wisconsin–Madison Madison WI 53705 USA

**Keywords:** agrin, blood–brain barrier, ischemia/reperfusion injury, ischemic stroke, positron emission tomography

## Abstract

Intrathecal injection, drugs transporting along perivascular spaces, represents an important route for maintaining blood–brain barrier (BBB) integrity after cerebral ischemia/reperfusion (I/R) injury. However, after being directly injected into cerebrospinal fluid (CSF), the temporal and spatial changes in the distribution of therapeutic protein drugs have remained unknown. Here, with positron emission tomography (PET) imaging, the uptake of ^89^Zr‐agrin is noninvasively and dynamically monitored. These data demonstrate the time–activity curve of drugs in the brain subregions and their spatial distribution in different organs after intrathecal administration. Furthermore, agrin treatment effectively inhibits BBB disruption by reducing the loss of tight‐junctional proteins. Importantly, the infarct volume is reduced; the number of apoptotic neurons is decreased; and neurological function is improved in mouse I/R injury models. Thus, intrathecal injection of agrin provides the basis for a new strategy to research and develop protein drugs for reducing the aggravation of I/R injury.

## Graphical abstract

Distribution of intrathecal injection of agrin and its roles in the blood–brain barrier in a mouse model of cerebral ischemia/reperfusion are discussed.

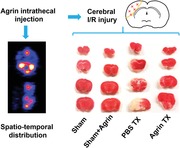

## 1 Introduction

Strokes are among the most common causes of disability and cause 1 of every 20 deaths in the USA, of which 87% are ischemic strokes.^1^ Blood–brain barrier (BBB) dysfunction is an essential pathological hallmark of ischemic strokes.[qv: 1b] Alteration of the extracellular matrix (ECM) is the main reason for BBB dysfunction, and directly affects the progression of ischemic stroke.^2^ In the ECM, agrin localization was observed in all basement membranes.^3^ Agrin is a large extracellular matrix heparan sulfate proteoglycan which contributes to the barrier properties of endothelial cells and stabilizing the tight connection in the BBB.^4^ Previous studies have demonstrated that agrin is also a key molecule in maintaining the integrity of ECM and modulating cell mobility.^5^ However, due to the low perfusion and capillary dysfunction during cerebral ischemia/reperfusion (I/R) injury, there has been an enormous challenge of delivering drugs to the ischemic penumbra via systemic administration.

Recently, the glymphatic system was discovered within brain that serves as a sink for exchange between cerebrospinal fluid (CSF) and interstitial fluid (ISF).^6^ However, there is still some controversy about the “glymphatic” function.^7^ The perivascular spaces (PVS), as the main component of glymphatic system, carry CSF through the brain, sweeping away metabolic waste.^8^ After being directly administrated into the CSF compartment, molecular imaging probes or contrast agents could move along the flow of CSF, and transport from the subarachnoid space entering the brain parenchyma via the PVS.^9^ However, for therapeutic proteoglycan, such as agrin, the distribution and residence time in different brain regions have been unknown.

Intrathecal (IT) drug delivery, as a method for bypassing the blood–brain barrier, has been used for managing chronic pain and spasticity.^10^ Due to the distribution of drugs around the blood vessels, intrathecal injection provides a precious way of protecting the BBB against cerebral I/R injury.^11^ In the study, we assumed that intrathecal injection of agrin could directly act on the blood–brain barrier by surrounding BBB. Positron emission tomography (PET) imaging was applied to noninvasively and dynamically monitor the biodistribution of agrin in vivo. Our data clearly showed the temporal and spatial changes in different brain regions after intrathecal injection. Importantly, intrathecal injection of agrin exhibited remarkable effects on inhibiting BBB disruption, which effectively relieved inflammatory damage and improved neuronal function. This study may inspire new perspectives for application of protein molecules the in fight against ischemic strokes.

## 2 Results

### 2.1 PET Imaging of Agrin In Vivo

**Figure 1:**
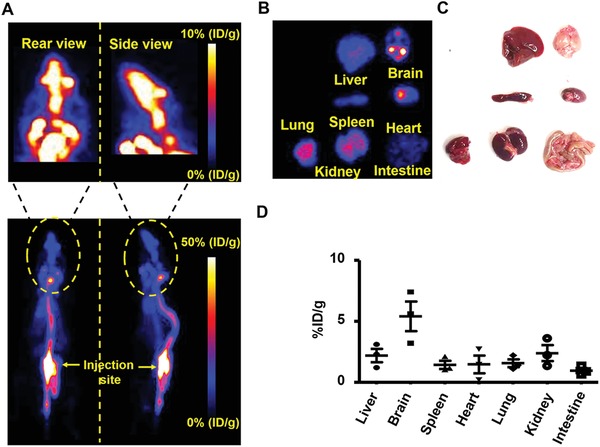
PET images of the biodistribution of agrin. A) Representative PET images of ^89^Zr–agrin in mice at 30 min after intrathecal injection. B) PET imaging of ^89^Zr–agrin in organs. C) Photograph of organs. D) Quantification of uptake of ^89^Zr–agrin in the heart, liver, spleen, lung, kidneys, intestine and brain at 30 min after intrathecal injection (mean ± SD, *n* = 3).

According to our previous study, the radionuclide ^89^Zr was used to label to agrin.^12^ To noninvasively investigate the biodistribution of agrin in vivo, PET imaging was performed with ^89^Zr–agrin at 30 min after injection. As shown in **Figure** **1**A, the signal from ^89^Zr–agrin in the spinal cord and brain was strong at 30 min post‐injection (p.i.). Furthermore, the horizontal and sagittal views of the brain PET images demonstrated that ^89^Zr–agrin transport via the spinal cord to the cisterna magna mainly distributes in the basilar artery and pituitary recess, then passes into the olfactory arterial complex and reaches the olfactory bulb. To investigate the biodistribution of agrin ex vivo, mice were sacrificed and scanned at 30 min p.i. As shown in Figure 1B,C, the signal from ^89^Zr–agrin was observed in the brain, heart, liver, spleen, lung, kidney, and intestines, suggesting that ^89^Zr–agrin returned to blood circulation following the flow of CSF within 30 min p.i. Quantitative ROI analysis of PET images showed that the uptake of ^89^Zr–agrin in the brain was significantly higher than other tissues (Figure 1D).

### 2.2 The Signal Change of ^89^Zr–Agrin within Brain

**Figure 2:**
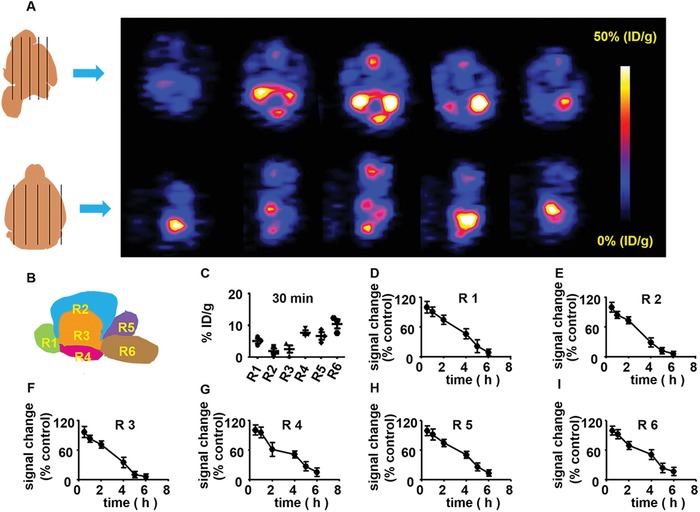
Representative brain PET imaging of ^89^Zr–agrin and signal change in mice. A) Different planes from representative PET imaging of ^89^Zr–agrin are shown at 30 min intrathecal injection after I/R. B) Schematic of sagittal midline section being divided six regions (Rs). Region 1 (R1) represents the olfactory bulb and anterior olfactory nucleus. Region 2 (R2) represents the cerebral cortex. Region 3 (R3) represents the hippocampus, caudate putamen, and thalamus. Region 4 (R4) represents the ventral striatum, basal forebrain, and hypothalamus. Region 5 (R5) represents the midbrain and cerebellum. Region 6 (R6) represents the pons medulla. C) The distribution of ^89^Zr–agrin within these six subregions at 30 min after intrathecal injection The signal change of D) R1, E) R2, F) R3, G) R4, H) R5, and I) R6 within 6 h after intrathecal injection.

To further observe the distribution of ^89^Zr–agrin within the different subregions of brain, the brain was removed at 30 min after intrathecal injection of ^89^Zr–agrin and then scanned from the horizontal and sagittal planes to obtain the various PET images (**Figure** **2**A). To investigate the distribution of ^89^Zr–agrin within different subregions of brain, the brain was subdivided into six regions (Figure 2B). Region 1 (R1) represents the olfactory bulb and anterior olfactory nucleus. Region 2 (R2) represents the cerebral cortex. Region 3 (R3) represents the hippocampus, caudate putamen, and thalamus. Region 4 (R4) represents the ventral striatum, basal forebrain, and hypothalamus. Region 5 (R5) represents the midbrain and cerebellum. Region 6 (R6) represents the pons medulla. To analyze the dynamic distribution within these subregions, manual delineation of ROIs was performed to obtain time–activity curves for ^89^Zr–agrin in the various subregions. The quantification of ^89^Zr–agrin within these six ROI subregions was performed at 30 min after intrathecal injection. In the study, the distribution of ^89^Zr–agrin was normalized to the values at 30 min p.i., and this was used to determine the signal change of ^89^Zr–agrin at 30 min, 1, 2, 4, 5, and 6 h after intrathecal injection. For all six subregions, the distribution of ^89^Zr–agrin began to decrease at 30 min almost to baseline until 6 h after intrathecal injection (Figure 2D–I).

### 2.3 The Accumulation of Agrin in the Penumbra

**Figure 3:**
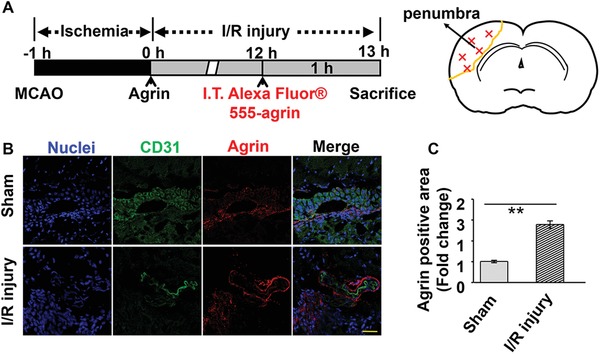
Biodistribution of Alexa Fluor 555–agrin. A) Time schedule for analysis of biodistribution of Alexa Fluor 555–agrin in the ischemic cortex. B) CD 31 (green) and Alexa Fluor 555–agrin (red) were detected by confocal microscopy in the ipsilateral cortex (scale bar, 50 µm). C) Quantitative analysis of Alexa Fluor 555–agrin in the ipsilateral cortex.

To investigate the accumulation and distribution of agrin in the penumbra, agrin was labeled with the Alexa Fluor 555 dye and measured after intrathecal injection (**Figure** **3**A). In normal mice, Alexa Fluor 555–agrin only accumulated within the PVS, and no Alexa Fluor 555–agrin was found in the brain parenchyma. In I/R injury mice, the structure of blood vessels (CD31‐labeled, green) shrunk and disappeared, and the extravasation of Alexa Fluor555‐labeled agrin was evident from labeled agrin diffusing into brain parenchyma (Figure 3B). Quantitative analysis of fluorescence images demonstrated that the larger area of Alexa Fluor 555‐labeled agrin was found in I/R injury mice, compared with sham group (Figure 3C).

### 2.4 The Role of Agrin on BBB Permeability in the Ischemic Penumbra

**Figure 4:**
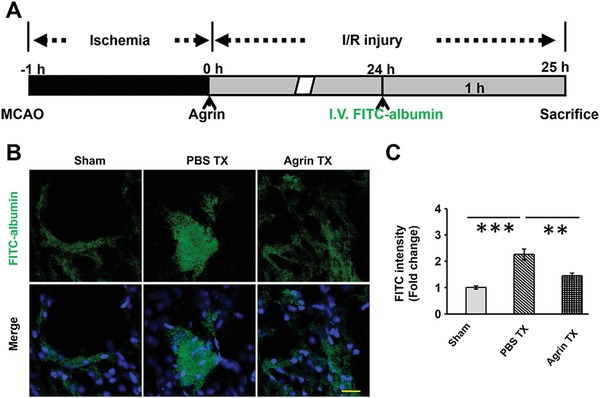
The role of agrin in BBB permeability in penumbra. A) Treatment schedule of mice. B) FITC–albumin detected by confocal microscopy in the ipsilateral cortex (scale bar, 25 µm). C) Quantitative analysis of FITC–albumin in the ipsilateral cortex.

To test the role of agrin in BBB permeability, mice in different groups were injected intravenously with FITC–albumin at 24 h after cerebral I/R, and their brains were removed at 25 h after cerebral I/R (**Figure** **4**A). In the sham group, the distribution of FITC–albumin was restricted to the lumen structure, suggesting that there is no leakage of FITC–albumin in a normal cerebral cortex. In the I/R group, mice showed a big area of leakage of FITC–albumin from the lumen of the vessels which diffused in the ischemic penumbra. Compared with the I/R group, significantly smaller diffusion area and lower fluorescence density were found in the agrin treatment (Tx) group (Figure 4B,C).

### 2.5 The Role of Agrin in Stabilizing the Tight‐Junction Proteins of the BBB

**Figure 5:**
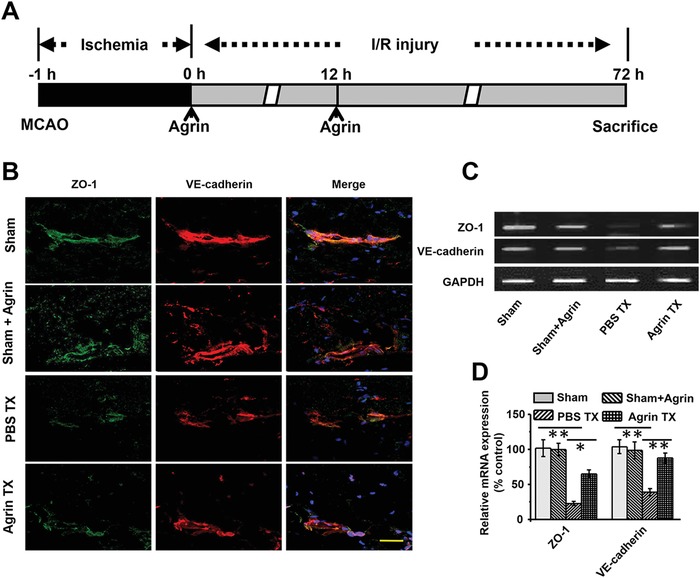
The role of agrin in stabilizing the tight‐junctional proteins of the BBB. A) Treatment schedule of mice. B) ZO‐1 and VE cadherin detected by confocal microscopy in the ipsilateral cortex (scale bar, 50 µm). The gene expression of ZO‐1 and VE cadherin was measured by PCR using C) gel electrophoresis and D) real‐time PCR in the ischemic penumbra (mean ± SD, *n* = 3, **p < 0.01).

To monitor the role of agrin Tx in stabilizing BBB integrity in the penumbra, agrin was intrathecally injected into the CSF at 0 and 12 h after reperfusion, followed by immunofluorescence staining for VE cadherin and zonula occludens‐1 (ZO‐1) in the BBB at 72 h after reperfusion (**Figure** **5**A). No significant difference in the expression of VE cadherin or ZO‐1 was found between the sham and sham + agrin groups. Compared with the sham groups, lower expression of VE cadherin and ZO‐1 was found in the phosphate buffered saline (PBS) Tx group. Importantly, there was higher VE cadherin and ZO‐1 expressions in the agrin Tx group compared to the PBS Tx (Figure 5B). In addition, higher VE cadherin and ZO‐1 gene expression were also detected in the agrin Tx group compared with the PBS Tx (Figure 5C,D). This suggests that agrin Tx could effectively inhibit the decrease of tight‐junctional protein expression on the BBB at 72 h after I/R injury.

### 2.6 Anti‐Inflammatory Effect of Agrin in the Ischemic Penumbra

**Figure 6:**
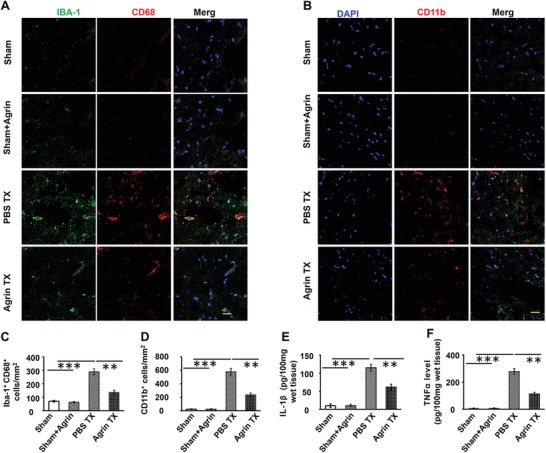
The agrin treatment inhibiting inflammation in the penumbra. Representative CD68 and A) Iba‐1 double‐positive cells and B) CD11b^+^ inflammatory in the penumbra; scale bar, 20 µm. Quantitative analysis of CD68 and C) Iba‐1 double‐positive cells and D) CD11b^+^ inflammation in the penumbra (mean ± SD, *n* = 12, **p < 0.01, ***p < 0.001). E) IL‐1β levels and F) TNF‐α levels in the ischemic penumbra were determined by ELISA (mean ± SD, *n* = 6, **p < 0.01, ***p < 0.001).

To study the anti‐inflammatory effect of agrin in the ischemic penumbra, we investigated the role of agrin in reducing the number of activated of microglia (Iba‐1^+^ and CD68^+^) and inflammatory cells (CD11b^+^ cells) at day 3 after I/R injury. Immunofluorescence staining and slice cell counting revealed that the number of CD68^+^/Iba‐1^+^ and CD11b^+^ inflammatory cells in the agrin Tx group was significantly lower than that in the PBS Tx group. No significant difference was found in the sham and sham with agrin groups (**Figure** **6**A–D). Enzyme‐linked immunosorbent assay (ELISA) assays also demonstrated that the concentrations of Interleukin‐1β (IL‐1β) and tumor necrosis factor‐α (TNF‐α) in the penumbra were lower in the agrin Tx case, compared with the PBS group, suggesting that agrin inhibits the release of proinflammatory factors in the penumbra at day 3 after I/R injury (Figure 6E,F).

### 2.7 Agrin Protecting Neurons Against I/R Injury

**Figure 7:**
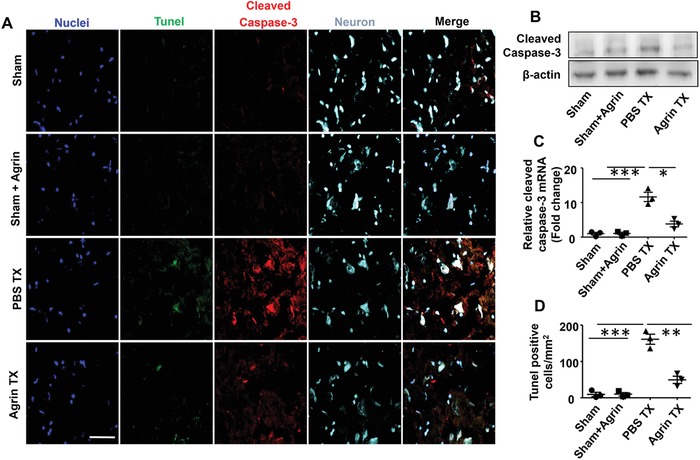
Agrin treatment protecting neurons at 72 h after I/R injury. A) Cleaved caspase‐3 (red), Tunel (green), and Nissl staining were measured by confocal microscopy in the ipsilateral cortex (scale bar, 50 µm). B) Activated caspase‐3 expression was measured by western blotting and C) real‐time PCR in the ischemic penumbra (mean ± SD, *n* = 3, **p < 0.01). D) Quantitative analysis of the Tunel‐positive cells (mean ± SD, *n* = 3, **p < 0.01, ***p < 0.001).

To further investigate the role of agrin in protecting neurons against I/R injury, mice received intrathecal injection of agrin. The expression of the cleaved form of caspase‐3 was measured by Nissl and Tunel staining in the ischemic penumbra. As shown in **Figure** **7**A, the number of Tunel positive cells and the expression of activated caspase‐3 were significantly increased in the PBS Tx group in relation to the sham groups. In contrast with the PBS Tx group, fewer activated caspase‐3 and Tunel positive cells were detected in the agrin Tx group. No significant difference in the gene and protein expression of activated caspase‐3 was found between the sham and sham with agrin groups. Higher levels of activated caspase‐3 expression were found in the PBS Tx compared with the sham group, which was effectively inhibited by agrin Tx on day 3 after I/R injury (Figure 7B,C). Tunel staining is an effective method for detecting DNA breakage. Significantly an increased number of tunel positive cells were found in the PBS Tx group, which was blocked by agrin Tx (Figure 7D). This indicates that agrin Tx could effectively protect neurons from dying during cerebral I/R injury.

### 2.8 The Effect of Agrin on Infarct Volume and Neurological Deficits

**Figure 8:**
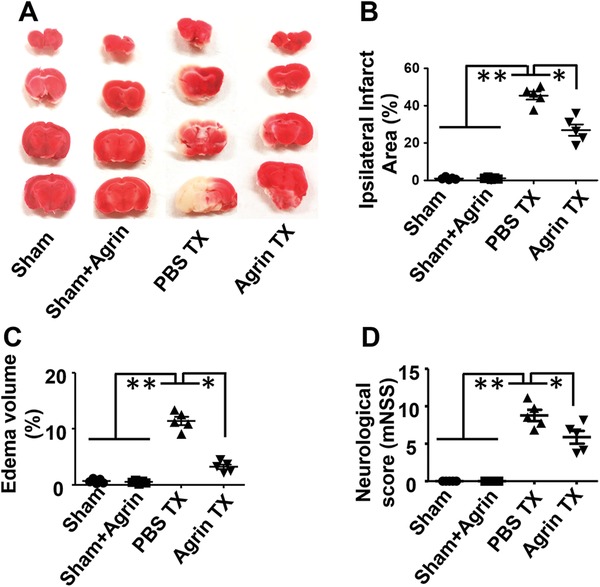
The agrin treatment attenuating brain injury at day 3 after cerebral I/R injury. A) Representative TTC staining, quantitative analysis of B) edema volume, C) infarct volume, and D) neurological scores in the different groups (*n* = 5, mean ± SD, *p <0.05, **p < 0.01).

To examine whether agrin Tx could reduce infarct volume on day 3 following I/R injury, TTC staining was performed to evaluate the infarct volume. No significant difference was found between the sham or sham treated with agrin groups, demonstrating the biocompatibility of agrin with the central nervous system (CNS). Compared with the PBS group, agrin Tx effectively reduced the infarct volume (**Figure** **8**A) and edema volume (Figure 8B,C). The mNSS was used to estimate neurological functions on day 3 after I/R injury. Agrin‐treated mice also showed lower mNSS scores compared to PBS‐treated mice, suggesting that agrin could effectively attenuate neurological deficits following cerebral I/R injury (Figure 8D).

## 3 Discussion

Proteins represent one of the most potential classes of therapeutics.^13^ Due to inhibition by the BBB, delivery of drugs into the brain is complicated and difficult.^14^ Intrathecal administration is a relatively straightforward approach of bypassing the BBB through drug injection into the CSF, as has been applied for spinal anesthesia, pain, or management of brain‐related disorders.^15^ In order to develop effective protein‐based drugs for the treatment of strokes, it is vital to use noninvasive methods to analyze the dynamic distribution of proteins along the CSF circulation. In this study, PET imaging clearly demonstrated that ^89^Zr–agrin moves along the CSF flow from the spinal cord into the brain parenchyma, and then reabsorbed into the blood circulation. Our data clearly demonstrated that the uptake of ^89^Zr–agrin peaked at 30 min after intrathecal injection, and gradually decreased over time to the baseline level at 6 h after intrathecal injection.

The glymphatic (glial‐lymphatic) pathway, representing one of the interstitial spaces of the brain, plays important roles on clearing extracellular metabolites and waste products from the parenchyma into the CSF.^16^ Previous studies have shown that different molecular weight contrast agents are transported along the external brain surface arteries and olfactory arteries within the subarachnoid space and move along these para‐arterial spaces into brain parenchyma.[qv: 9b,17] To investigate the distribution of agrin in the PVS, Alexa Fluor555–agrin was measured by confocal microscopy. The collapsed and shrunk arterial structure and the extravasation of Alexa Fluor555‐agrin were also found in I/R mice. This demonstrated that I/R injury resulted in glymphatic dysfunction and structural damage,^18^ which is closely associated with agrin exudation from the PVS and diffusion into the brain interstitium.

Agrin, as a heparan sulfate proteoglycan, exhibits multiple protective effects against some diseases, such as anti‐inflammatory^19^ and regulating neuromuscular synapse formation.^20^ The decreased expression of agrin is correlated with the loss of tight‐junctional proteins or with enhanced BBB leakiness in brain‐related diseases.[qv: 3a,21] Due to transport within the glymphatic pathway, agrin can act directly on the brain vascular basement membranes, capillary endothelial cells (BCECs), pericytes, and astrocytes of the BBB. Therefore, intrathecal injection of agrin has great potential to protect the BBB from disruption during I/R injury.

Previous studies demonstrated^22^ that BBB permeability is closely related to edema and hemorrhagic transformation during I/R injury. The expression of tight‐junctional proteins plays important roles in maintaining the integrity of the BBB. FITC–albumin has been used to evaluate the BBB permeability for brain diseases. Here, our results demonstrated that agrin Tx could reduce the FITC–albumin leakage in the ischemic penumbra, due to the intrathecal injection of agrin and the ability to maintain the BBB integrity by inhibiting the loss of tight‐junctional proteins.

During cerebral I/R injury, BBB disruption is closely related to the extent of inflammation in the penumbra.^23^ M1 microglia represent the proinflammatory microglia, and their major surface markers included the ionizing calcium‐binding adaptor molecule 1 (Iba‐1) and CD68.[qv: 22c,24] In addition, polymorphonuclear neutrophils play a key role in inflammatory damage after I/R injury, and CD11b^+^ is a major surface marker.^25^ After brain I/R injury, M1 microglia and neutrophils are rapidly recruited into the ischemic penumbra, which enhances the release of proinflammatory factors.^26^ Our results demonstrated that agrin Tx could effectively reduce the accumulation of activated microglia and neutrophils and inhibit the release of proinflammatory factors in the ischemic penumbra at 72 h after I/R injury. This suggested that agrin Tx could prevent secondary inflammation from I/R injury.

Tunel staining is a classic method for detecting apoptotic cells via analyzing DNA degradation during the late stages of apoptosis.^27^ The cleaved form of caspase‐3 has been used as a key mediator of neuronal apoptosis.^28^ Nissl staining, as a classic method, has been used for staining neurons in brain sections. In this study, our results clearly demonstrated that agrin treatment could effectively reduce the number of Tunel positive cells and activated caspase‐3/Nissl double‐positive neurons in the ischemic penumbra. These results indicated that neuronal apoptosis could be effectively decreased by agrin Tx.

During cerebral I/R injury, the BBB disruption plays a decisive role in edema, infarcted volume, and neurological dysfunctions.^29^ Based on TTC staining and quantitative analysis of ROI exhibiting edema, we found that agrin treatment could effectively reduce the infarcted volume and edema at 72 h after I/R injury. The mNSS has been used to estimate neurological functions after brain injury.^24^ Our results also demonstrated that agrin Tx could effectively improve the neurological function in mice with I/R injury.

## 4 Conclusions

In conclusion, we quantified the distribution of ^89^Zr–agrin within different subregions of the brain after intrathecal injection and found that the distribution of agrin around the BBB could protect the BBB integrity against I/R injury. We confirmed that intrathecal injection could be used as a potential and efficient route. In this context, innovative imaging methods including magnetic resonance imaging of adhesion molecules may effectively and noninvasively detect this inflammatory penumbra and thus be able to select patients eligible for such therapy. Furthermore, the agrin Tx could reduce infarcted volume and improve neurological function in mouse I/R injury models. Therefore, intrathecal injection is an excellent route for using agrin to treat cerebral I/R injury.

## 5 Experimental Section

*Materials*: The neural agrin was purchased from Sigma‐Aldrich Corp. (R&D Systems). Western blotting and immunofluorescence staining were carried out with the following primary antibodies: anti‐Iba‐1, anti‐CD‐86, anti‐occludin, anti‐ZO1, and anti‐caspase‐3 antibodies, which were purchased from Abcam (Cambridge, UK). Secondary antibodies used in this study include Alexa Fluor 488‐conjugated and 594‐conjugated. Ultrapurified water was prepared using a Milli‐Q Synthesis System (Millipore, Bedford, MA).

*Polymerase Chain Reaction and Quantitative Polymerase Chain Reaction*: For polymerase chain reaction (PCR) or quantitative polymerase chain reaction (qPCR) analysis, total RNA was isolated from the ischemic penumbra using RNeasy mini‐kits (Qiagen, Duesseldorf, Germany), and was transcribed into cDNAs by the PrimeScript 1st Strand cDNA Synthesis Kit (Takara, Otsu, Japan). The FastStart Universal Probe Master (Roche Applied Science, Indianapolis) was used for real‐time PCR. Amplification conditions were 30 cycles of denaturation at 95 °C for 30 s, annealing at 55 °C for 30 s, and extension at 72 °C for 30 s. Sequences of the primers used in the present study were as follows: VE cadherin, forward primer (5′‐TCTTGCCAGCAAACTCTCCT‐3′) and reverse primer (5′‐TTGGAATCAAATGCACATCG‐3′); ZO‐1 forward primer (5′‐CCACCTCTGTCCAGCTCTTC‐3′) and reverse primer (5′‐CACCGGAGTGATGGTTTTCT‐3′); and mouse glyceraldehyde‐3‐phosphate dehydrogenase (GAPDH) (forward: 5′‐AGAGGGAAATCGTGCGTGAC‐3′ and reverse: 5′‐CAATAGTGATGACCTGGCCGT‐3′). Relative messenger ribonucleic acid expression of the target genes was normalized to the expression of GAPDH.

*Radiolabeling of Agrin*: For radiolabeling of agrin with ^89^Zr, *p*‐SCN‐Bn‐deferoxamine (DFO) was conjugated to agrin through the exposed lysine residues.^30^ Briefly, 4 mg of agrin (in PBS) was incubated with DFO (in dimethyl sulfoxide) at a molar ratio of 1:10 (agrin:DFO) at room temperature for 2 h. ^89^Zr–DFO–agrin was purified, collected, and filtered before injection into mice.

*PET Imaging of Agrin in Mice*: ^89^Zr‐labeled agrin was injected intrathecally into mice with cerebral I/R injury. A Siemens Inveon microPET/CT (Siemens Medical Solutions, Erlangen, Germany) was used for longitudinal PET scans at 30 min, 1, 2, 4, 5, and 6 h after injection. Region‐of‐interest (ROI) data were generated from counts/pixel and the region volume for determining time–activity curves in different organs or subregions of brain. The data were then normalized to the injected dose to acquire the final ROI signal in %ID g^−1^.

*Measurement of FITC–Albumin Extravasation*: To detect BBB breakdown, fluorescein isothiocyanate (FITC)–albumin (2 mg in 0.1 mL saline) was injected by tail vein. FITC–albumin was injected at 24 h after I/R injury, and the animals were euthanized and their brains removed at 25 h after I/R injury. A Nikon A1RS confocal microscope and the standard computer‐assisted image analysis technique were used for detecting the FITC–albumin positive areas. For quantitative analysis of FITC–albumin leakage, the protein lysates were required from ipsilateral brain tissue (covering bregma from −2.06 to 1.18 mm). The FITC–albumin leakage was determined by measuring FITC fluorescence intensity of the protein lysates. FITC–albumin intensity was determined using a standard curve.

*Tissue Immunostainings*: After being fixed with 4% paraformaldehyde for 24 h, brains were cut into coronal slices (12 µm), then incubated with 10% horse serum for 1 h at room temperature. These sections were then incubated with monoclonal anti‐Iba‐1 (1:100 in PBS with 1% bovine serum albumin (BSA)), anti‐CD11b (1:100 in PBS with 1% BSA), anti‐CD68 (1:100 in PBS with 1% BSA), and anticleaved caspase‐3 antibodies (1:100 in PBS with 1% BSA) for 2 nights at 4 °C at night, and then were washed and incubated with secondary antibodies for 1 h. Nissl staining was counterstained with anticleaved caspase‐3 and anti‐superoxide dismutase antibodies. Images were captured using a Nikon A1RS confocal microscope. Three brain coronal sections per mouse (+1.18, −0.10, and −2.06 mm from bregma, George Paxinos, and Keith B. J. Franklin, The Mouse Brain in Stereotaxic Coordinates, Academic Press) were used to quantify the stained area. The number of Iba^+^/CD68^+^ and CD11b^+^ cells per mm^2^ within the areas were measured from ten different images (ImageJ, National Institutes of Health).^31^

*Enzyme‐Linked Immunosorbent Assay*: After brain tissues had been homogenized in RIPA buffer (10 µL mg^−1^ brain), the protein concentration was quantified Protein Assay kit (Pierce, Appleton, WI, USA). According to the ELISA kit's instructions (MyBioSource, San Diego, USA), the concentrations of IL‐1β and TNF‐α were calculated from the standard curve for each experiment which was used for quantitative analysis of IL‐1β and TNF‐α level in the ischemic penumbra.

*Cerebral I/R Injury Model in Mice*: C57BL/6J male mice (26–28 g, Beijing HFK Bioscience Co., Ltd) were cared for and used according to the Guide of the Care and Use of Laboratory Animals, and the procedure was approved by the Animal Care and Use Committee of the University of Nanchang. The cerebral I/R injury model was induced using the intraluminal filament model with minor modifications.^32^ After mice were anesthetized with inhaled isoflurane, a midline neck incision was created to expose the right carotid artery. The 4–0 nylon suture with silicon‐coated tip was inserted from the left external carotid artery into the left internal carotid artery and advanced to the circle of Willis. Following 60 min of occlusion, the nylon suture was withdrawn to establish reperfusion.

*Evaluation of Neurological Deficits and Infarct Size*: Mice were randomly divided into four groups: the sham group, sham group with agrin (0.2 mg mL^−1^), I/R model mice treated with PBS (PBS group), and I/R model mice treated with agrin (0.2 mg mL^−1^). Mice were treated with 10 µL agrin twice via separate intrathecal injections at 0 and 12 h after the onset of I/R injury. The modified Neurological Severity Score (mNSS) includes motor, sensory, reflex, and balance tests with a total score of 18. A score of 0 represents normal, with a higher score indicative of more significant injury. At days 3 after I/R injury, the animals were euthanized, and their brains were removed and stained with 2% 2,3,5‐triphenyl tetrazolium chloride staining (TTC, Sigma, UK). The infarct area and the edema volume were evaluated using an image analysis system (ImageJ software, NIH).

*Statistical Analysis*: Data were expressed as mean ± standard deviation (SD). Student's *t*‐test (for two groups) or one‐way analysis of variance (ANOVA) (for three or more groups) was used to determine statistically significant differences between experimental and control samples. In all analyses, statistical significance was set at a *p*‐value of <0.05.

## Conflict of Interest

The authors declare no conflict of interest.
